# Supplementary material for: A comparison of software for analysis of rare and common short tandem repeat (STR) variation using human genome sequences from clinical and population-based samples
Source: PLoS One. 2024 Apr 1;19(4):e0300545. doi: 10.1371/journal.pone.0300545 (PMC10984476; doi:10.1371/journal.pone.0300545)
Supplement: S2 Table — (DOCX) [file pone.0300545.s002.docx]

**S2 Table. Number of loci called as percentage of total in catalogue for GangSTR and HipSTR and ExpansionHunter, and call concordance as function of sequence length**

| Tool | Sample | No of loci in catalogue bed file | No. of calls at 2x150 bp | No. of calls at 2x250 bp | Calls in both | Identical by allele length (%) | Identical by allele sequence (%) |
| --- | --- | --- | --- | --- | --- | --- | --- |
| HipSTR.v0.6.2 | HG002 | 790661 | 96.1 | 94.1 | 93.5 | 99.6 | 99.8 |
|  | HG003 | 790661 | 96.1 | 93.8 | 93.8 | 99.6 | 99.8 |
|  | HG004 | 790661 | 95.8 | 93.9 | 93.5 | 99.6 | 99.8 |
|  |  |  |  |  |  |  |  |
| GangSTR.v2.5.4 | HG002 | 790661 | 99.7 | 96.1 | 96.1 | 99.4 | x |
|  | HG003 | 790661 | 99.7 | 95.8 | 95.8 | 99.4 | x |
|  | HG004 | 790661 | 99.2 | 96.8 | 96.8 | 99.4 | x |
|  |  |  |  |  |  |  |  |
| ExpansionHunter.v5.0.0 | HG002 | 790661 | 99.8 | 99.3 | 99.3 | 97.6 | x |
|  | HG003 | 790661 | 99.8 | 99.8 | 99.8 | 97.5 | x |
|  | HG004 | 790661 | 99.3 | 99.4 | 99.3 | 97.3 | x |

X – not analysed
